# Posterior paramedian approach combined with a novel inverted V-shaped surgical access for intraspinal schwannomas: a retrospective case series study

**DOI:** 10.1186/s13018-023-03816-3

**Published:** 2023-05-15

**Authors:** Pengfei Zhai, Haiyang Wu, Linjian Tong, Yulin Wang, Zhiming Sun

**Affiliations:** 1 Department of NeuroSpine Surgery, Tianjin Huanhu Hospital, Tianjin, China; 2 Clinical College of Neurology, Neurosurgery and Neurorehabilitation, Tianjin Medical University, Tianjin, China; 3 Duke Molecular Physiology Institute, Duke University School of Medicine, Duke University, Durham, NC USA

**Keywords:** Intraspinal schwannomas, Microsurgical surgery, Posterior paramedian approach, Inverted “V” shaped

## Abstract

**Objective:**

To explore the efficacy and safety of the posterior paramedian approach combined with a novel inverted V-shaped surgical access for the treatment of intraspinal schwannomas.

**Methods:**

This study retrospectively reviewed consecutive patients who underwent surgical resection of the intraspinal schwannomas via the inverted V-shaped approach at our center between January 2016 and May 2021. Changes between the preoperative and postoperative visual analog scale (VAS) scores and neurological function Japan Orthopaedic Association (JOA) scores were assessed. Secondary outcomes such as success rate of tumor resection, operation time, blood loss, spinal stability, and disruption degree of intervertebral joints. Postoperative complications were also investigated.

**Results:**

Of these 36 consecutive patients, there were 6 cases in the cervical spine, 2 cases at the cervical-thoracic junction, 11 cases in the thoracic spine, 4 cases at the thoracic-lumbar junction and 13 cases in the lumbar spine. The average operation time was 99 min, and the average blood loss was 95.4 mL. The tumor removal rate was 100%. Postoperative CT re-examination showed that the spinous processes were intact in all cases, the facet joint surfaces were intact in 32 cases. At the time of last follow-up, the median JOA score was 25 (9–27), which was significantly improved compared to the preoperative median JOA score of 15 (10–22) (*P* < 0.01). The overall excellent and good rate were 88.9 %. The median VAS score at post-surgery was 0 (0–2), which was significantly improved compared to the preoperative median VAS score of 4 (2–8) (*P* < 0.01). As for complications, there were no cases of cerebrospinal fluid leakage or spinal instability. Three patients who had a postoperative fever finally recovered after lumbar cistern drainage.

**Conclusion:**

The inverted V-shaped surgical access via the posterior paramedian approach is an effective and safe method for the treatment of intraspinal schwannomas.

## Introduction

Schwannomas, also known as the nerve sheath tumors, neurinomas or neurilemmomas, are the most common type of intraspinal tumors, which are frequently observed in the cervical and lumbar regions [1]. According to statistics, the incidence of intradural primary tumors is approximately 0.74 per 10,000 per year, with 25% of these being schwannomas [2]. The symptoms of intraspinal schwannomas may include radicular pain, numbness, motor weakness caused by nerve root stimulation and also walking disability, incontinence due to spinal cord compression. At present, it is commonly assumed that surgical resection is the most effective treatment option [1, 3, 4]. The classic procedure is a posterior paramedian approach combined with total-laminectomy for removal of schwannomas. Nevertheless, with the recent progression of minimally invasive spine surgery, a posterior paramedian approach with unilateral laminectomy via fenestration has emerged [5, 6]. A large series of spinal tumors utilizing unilateral laminectomy or total-laminectomy drew the conclusions that juxtamedullary tumors of benign pathology were ideal choice for unilateral laminectomy, while complicated malignant tumors with multiple etiologies required a full laminectomy for sufficient surgical field and resection [7].

However, with the rapid popularization of these surgical approaches, the shortcomings of these methods have been gradually revealed. Take the total laminectomy as an example, several scholars have reported spinal deformity and instability as common complications after total laminectomy to remove spinal tumors [8–10]. The posterolateral approach with tubular retractor and laminectomy window is limited by the tumor diameter, and it is difficult to obtain surgical field view when the tumor diameter is larger than the tubular retractor. Therefore, further investigations are quite necessary to develop new approach for intraspinal schwannomas. In recent years, on the basis of the classical surgical approaches and taking into account the anatomy and pathophysiological mechanism of intraspinal schwannomas, we have applied a posterior approach combined with a novel inverted V-shaped surgical access for intraspinal schwannomas. The aim of this retrospective study was to report our experience on this approach and evaluate the clinical results.

## Materials and methods

### General information

This study was approved by the ethics committee of the Tianjin Huanhu Hospital. Patients with intraspinal schwannomas undergone posterior paramedian approach combined with inverted V-shaped surgical access between January 2016 and May 2021 were retrospectively analyzed. The inclusion criteria were that (1) intraspinal schwannoma patients were confirmed by physical examination, imaging examination including X-ray, computed tomography (CT), and magnetic resonance imaging (MRI), and postoperative pathology; (2) the greatest tumor diameter did not exceed 5 cm; (3) patients were no older than 75 years old; (4) length of follow-up greater than 1 year. Exclusion criteria: (1) other intraspinal tumor types; (2) tumors located outside the spinal canal; (3) patients who had a surgical history at the same vertebral level; (4) periodical follow-up data were incomplete.

### Surgical method

**Fig. 1:**
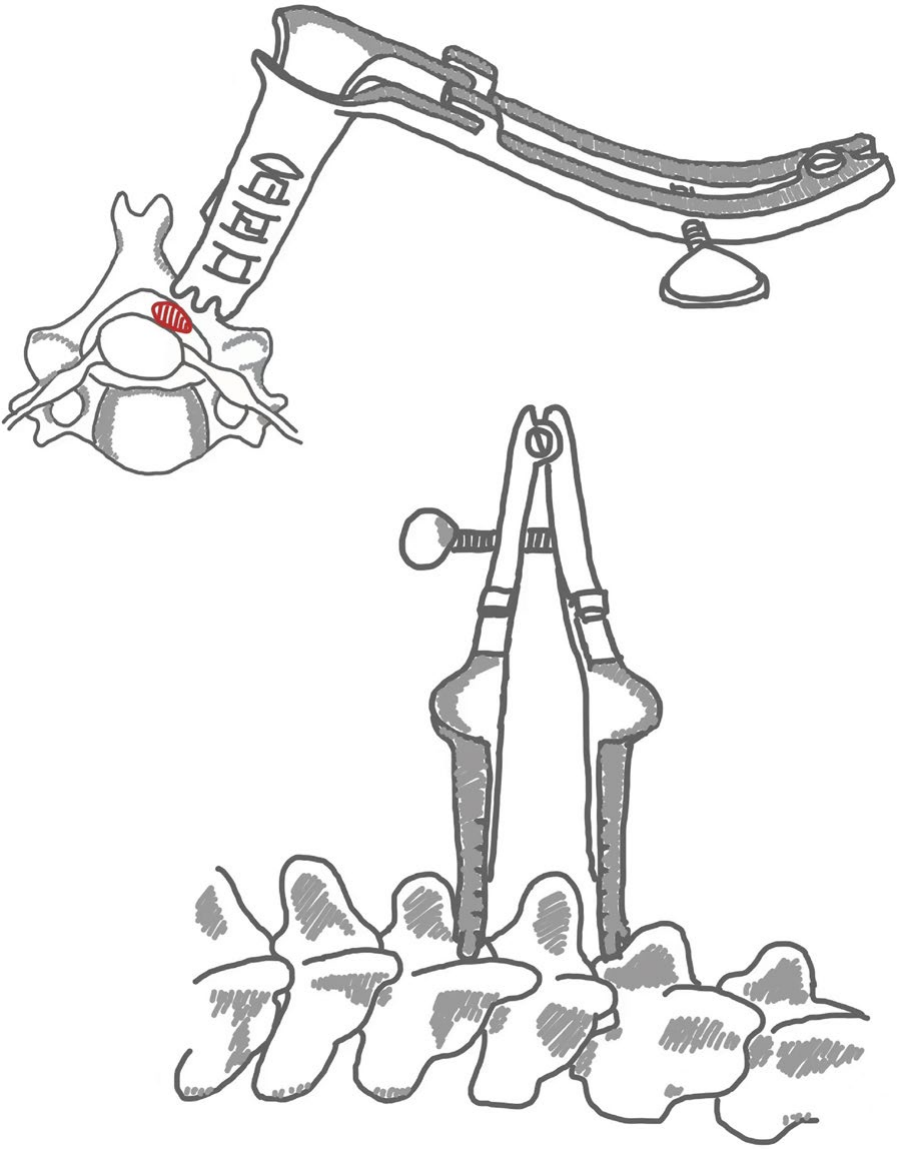
Schematic of the special retractor for inverted V-shaped surgical access

Patients under conventional tracheal anesthesia were placed in the prone position. A head-frame was used for the neck surgery, whereas a latex cushion was used for the thoracic and lumbar regions. Electrophysiological monitoring was performed during the surgery and the lesion segment was determined using a C-arm fluoroscope. At the level of the lesion segment, 2–3 cm lateral to the midline of the spine, a 3–4 cm longitudinal incision was made. The incision was positioned towards the left or right side of the spinal canal based on the location of the tumor. The skin, subcutaneous fascia and deep fascia were consecutively cut open. The paravertebral muscles were separated until the exposure of target lamina. Then place a special retractor (Medtronic Inc. Fig. 1), with the lower end of the retractor reaching the vertebral plate, and retract both upwards and downwards, forming an inverted “V” shape. The range of expansion of this retractor was determined based on the size of the tumor. The soft tissue in the surgical area is then stripped again using a monopolar electrocautery, reaching the root of the transverse process on the inside and the joint process on the outside. Under the microscope, the target laminae were drilled open, partially or completely depending on the size of the tumor. Afterwards, the ligamentum flavum was resected, and the dura was cut along the midline and suspended with thin wire. The adhesions around the tumor were stripped and the parent nerve was cut. Then the tumor was removed piecemeal until completely resected. The removal of the intra-foraminal portion was performed using a hook and hook extraction. Finally, dural sac was closely and continuously sutured. A watertight dural closure was subsequently performed with the artificial dura. Artificial bone was implanted around the articular processes and the vertebral defect place. No drainage tube was placed post-operatively.

### Postoperative management

A prophylactic antibiotic (Cefuroxime) was intravenously used to prevent infection. Patients were also dehydrated (dehydration, hormone) to reduce cerebral edema. On the fourth postoperative day, the patients began early ambulation with the protection of a brace. Before discharging from hospital, all patients were re-examined with MRI and CT scan to evaluate the extent of tumor resection. Follow-up was performed by phone calls or outpatient review. At each follow-up time point (1-month, 2-month, 3-month, 6-month and the latest follow-up), adverse events, clinical and radiographic evaluation were conducted by the senior author.

### Evaluation criteria

Visual analogue scale (VAS) and Japanese Orthopedic Association (JOA) score were used to evaluate functional status preoperatively. The VAS score is 10 points in total, where 0 points indicate no pain; 0–3 points indicate mild pain that can still be tolerated; 4–6 points indicate moderate pain that affects sleep; 7–10 points indicate a trend of increasing pain that is difficult to tolerate, most of which are accompanied by sleep disorders and passive positions. This score is commonly used to evaluate the patient’s preoperative and postoperative pain levels, with higher scores indicating more severe pain [11]. The JOA score is a total of 29 points, including four major aspects: clinical symptoms, signs, degree of daily activity restriction, and bladder function. This score is commonly used to evaluate the neurofunctional status of patients with spinal disorders before and after surgery, with higher scores indicating less impact on the patient's neurofunction [12]. Additionally, the improvement rate = (postoperative scores − preoperative scores)/(29 − preoperative scores) × 100%. The improvement rate was categorized as excellent (≥ 75%), good (74–50%), fair (49–25%), or poor (< 25%). The judgment criteria of spinal instability were that the change of spinal curvature at the surgical segments more than 10º under anteroposterior X-ray or more than 15º under lateral X-ray.

### Statistical analyses

Statistical analysis was performed using the Statistical Package for the Social Sciences software (version 21.0, USA). Descriptive statistics were used to describe demographics and clinical characteristics. All continuous variables were firstly tested for normal distribution by using Kolmogorov–Smirnov test. Variables with normal distribution were expressed as mean ± SD, or median (range) without normal distribution. The continuous variables that did not conform to a normal distribution were examined using non-parametric tests.

## Results

### Patients’ information

Table 1 summarized the basic information of all patients. A total of 36 cases with 22 males and 14 females, ranging in age from 19 to 70 years old were included. There were 6 cases in the cervical spine, 3 of which communicated with outside the spinal canal; 2 cases at the cervical-thoracic junction; 11 cases in the thoracic spine, 4 of which communicated with outside the spinal canal; 4 cases at the thoracic-lumbar junction; 13 cases in the lumbar spine. The average maximum tumor diameter was 3.2 cm (range 2.1 to 4.8 cm).

**Table 1:** The demographic data and perioperative outcomes

Indicators	Value
Age (year, mean ± SD)	48.0 ± 15.0
*Gender (n, %)*	
Male	22 (61.1)
Female	14 (38.9)
*Tumor site (n, %)*	
*C*	6 (16.7)
*C*-*T*	2 (5.6)
*T*	11 (30.6)
*T*-*L*	4 (11.1)
*L*	13 (36.1)
*Communicated with outside (n, %)*	
Yes	7 (19.4)
No	29 (80.6)
Maximum tumor diameter (cm, mean ± SD)	3.2 ± 0.8
Operation time (min, mean ± SD)	99.0 ± 25.3
Blood loss (ML, mean ± SD)	95.4 ± 33.7
Follow-up time (month, mean ± SD)	23.5 ± 13.1
Preoperative JOA (mean ± SD)	14.6 ± 2.7
Preoperative VAS (mean ± SD)	4.3 ± 1.9
Postoperative JOA [median (range)]	25 (9–27)
Postoperative VAS [median (range)]	0 (0–2)

VAS, visual analogue scale; JOA, Japanese Orthopedic Association

### Operative details

**Fig. 2:**
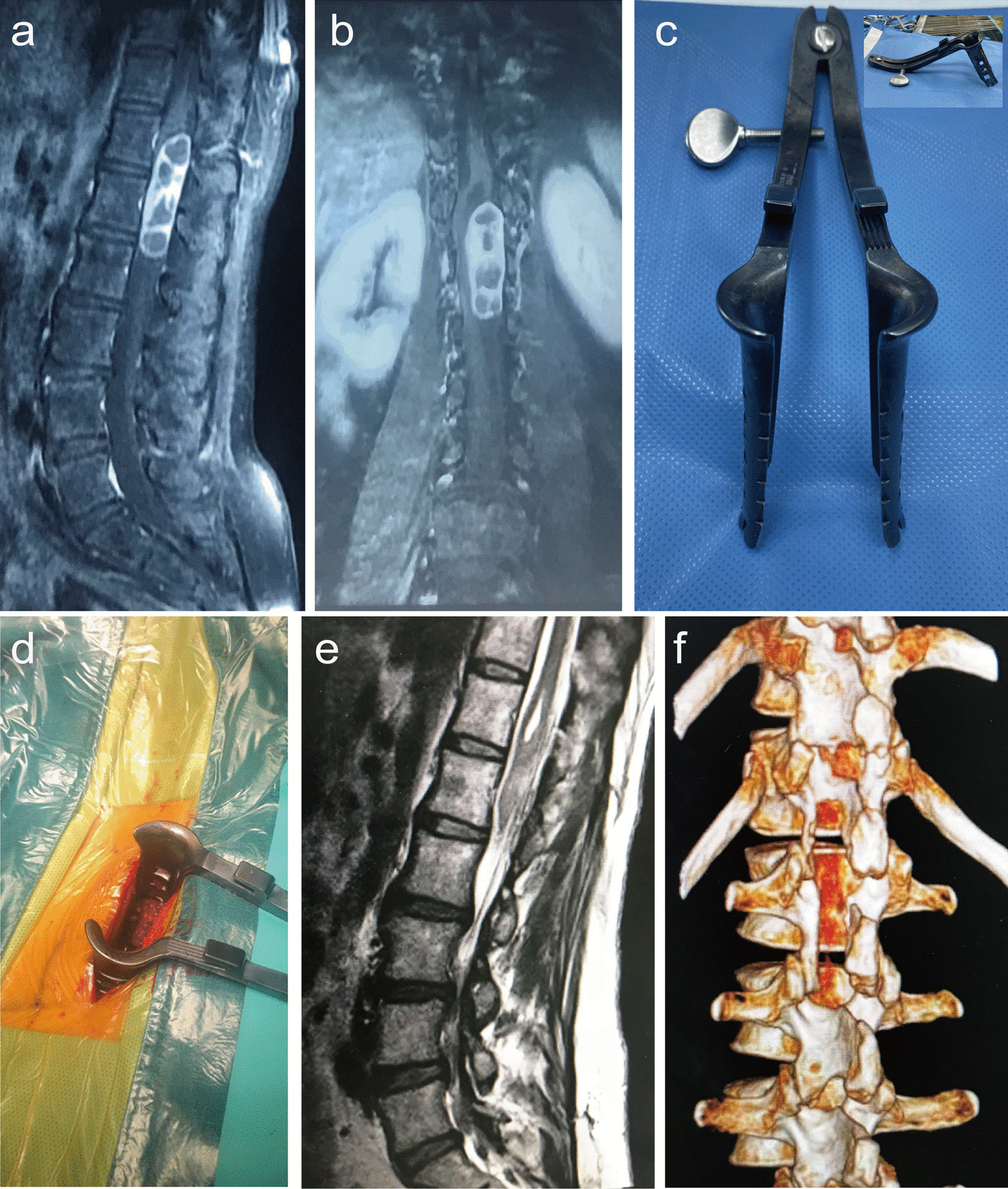
Typical case: **a** A 51-year-old woman presented with low back pain and numbness in her left lower limb for 1 year. Postoperative pathological diagnosis was intraspinal schwannoma. Physical examination showed hypoaesthesia in lateral side of the left leg and dorsum of feet. **a**, **b** MRI indicated a space-occupying lesion from T12 to L2 transpinal canal. **c** Real-object picture of the special retractor. **d** Posterior paramedian approach combined with inverted V-shaped surgical access. **e**, **f** Postoperative X-ray and three-dimensional reconstruction CT suggested that the tumor tissue was completely resected and intervertebral joint was damaged in a small range

All patients underwent surgical treatment through a posterior paramedian approach combined with inverted V-shaped surgical access. The average operation time was 99.0 ± 25.3 min, and the average blood loss was 95.4 ± 33.7 mL. During the operations, there was no damage to the spinous processes or interspinous ligaments in any of the 36 patients. Among the 32 patients with intradural extramedullary tumors, the vertebral foramen and facet joints were intact. In the 9 cases where the tumors extended into the vertebral foramen, the tumor was removed through the spinal canal, and the intervertebral foramen was completely or partially preserved on the side of the surgery. One week after surgery, a follow-up examination using three-dimensional reconstructed CT scans of the spine was conducted. The results showed that the spinous processes were intact in all cases, the facet joint surfaces were intact in 32 cases, and partially damaged in two cases. The tumor removal rate was 100%. A typical case was shown in Fig. 2.

### Follow up and functional scores

**Fig. 3:**
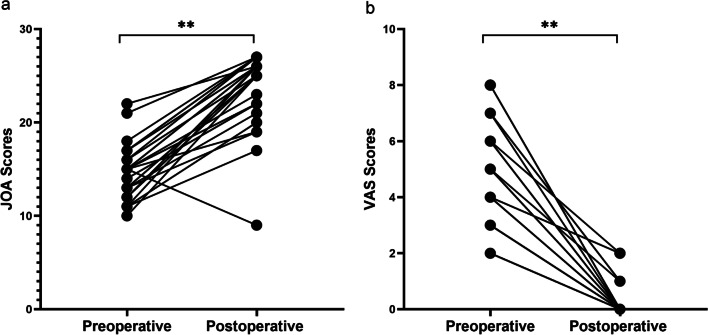
**a** Comparison of preoperative and postoperative JOA scores; **b** comparison of preoperative and postoperative VAS scores

All 36 patients were followed up for more than one year with an average follow-up duration of 23.5 months. During the follow-up, the JOA and VAS scores were used to assess the recovery of the patients. The results showed that at the time of last follow-up, the median JOA score was 25 (9–27), which was significantly improved compared to the preoperative median JOA score of 15 (10–22) (*P* < 0.01) (Table 1, Fig. 3a). One patient at the T5-6 level had a postoperative decrease of JOA score to 6 points, and had a 3-point improvement at 12 months post-surgery. According to the formula of improvement rate, 20 cases (55.6%) were graded as excellent, 12 cases (33.3%) as good, and 3 cases (8.3%) as fair, and one case (2.8%) as poor. The overall excellent and good rate were 88.9%. In addition, the median VAS score at post-surgery was 0 (0–2), which was significantly improved compared to the preoperative median VAS score of 4 (2–8) (*P* < 0.01) (Table 1, Fig. 3b). In addition, no tumor recurrences or spinal instability were observed throughout the follow-up period.

### Complications

After surgery, there were no cases of cerebrospinal fluid leakage. Three patients had a postoperative fever above 38 °C, and underwent lumbar cistern drainage for 5–7 days. Finally, their body temperature returned to normal and cerebrospinal fluid cultures indicated no bacterial growth. All incisions after surgery were stage I healing.

## Discussion

Schwannomas are usually benign encapsulated tumors of neurogenic origin arising from the cells forming the neural sheath. Therefore, schwannomas may occur anywhere along the spinal nerve [1–3]. The tumor tissues are mostly round or oval and vary in size. Previous studies have reported that this disease mainly affected individuals aged 20–60 years without peak age or sex predisposition [13, 14]. It predominantly involves lumbar, thoracic, lumbosacral, and cervical in the order of frequency of occurrence [15]. In this case series, there were 6 cases in the cervical spine, 2 cases at the cervical-thoracic junction, 11 cases in the thoracic spine, 4 cases at the thoracic-lumbar junction and 13 cases in the lumbar spine. This observation is generally consistent with the results reported in previous literatures [15, 16]. Generally speaking, low back pain and radicular pain are the most common initial symptoms for schwannomas. In our study, 30 cases developed radicular pain as the initial symptom. Then eventually develop symptoms of cord compression such as progressive limb stiffness and weakness, walking disturbance, and sphincter dysfunction. Therefore, the physical examination could roughly determine the lesion segments.

It was shown that schwannomas are less sensitive to radiotherapy and chemotherapy. Surgical excision should be performed once diagnosed, especially in symptomatic cases [17]. The aims of surgery are complete tumor excision, relieve spinal cord compression, improve neurological function and long-term quality of life. Generally, the choice of surgical methods should depend on the location and extent of tumor invasion. The classic surgical approach for intradural schwannomas is the total laminectomy via posterior paramedian approach [18, 19]. However, in recent years, this approach has been gradually replaced with the development of minimally invasive techniques. The main reasons are as follows: Firstly, the total laminectomy via posterior paramedian approach is highly traumatic and requires the vertical incision of bilateral paraspinal muscles and spinous processes, which are often the starting or ending points of the muscles adjacent to the spine. Even with careful and effective suturing and good healing, this is still a form of invisible and permanent damage to the muscles. The interspinous ligament plays an important role in stabilizing the posterior column of the spine, and this surgical approach could damage it, thereby affecting the stability of the posterior column [8–10]. Secondly, multiple studies have reported that after the total laminectomy, there can be postoperative kyphosis, especially in the cervicothoracic, thoracolumbar junction, or long-segment resections [20]. Many long-term follow-up studies showed that the formation of kyphosis should not be ignored, and there may even be a recurrence of spinal cord compression, which required corrective surgery to relieve the spinal cord compression. Thirdly, the frequently occurring cerebrospinal fluid leakage is another important reason. It is extremely difficult to achieve complete airtight closure after dura mater incision, and to prevent cerebrospinal fluid leakage, the fascia needs to be sutured tightly, rather than the muscles [8–10]. If the muscles are sutured too tightly, complications such as muscle fiber necrosis may occur. Therefore, the handling of the fascia is particularly important in avoiding postoperative cerebrospinal fluid leakage. In comparison to the cervical spine, the fascia of the lumbar spine is weaker and may result in insufficient suturing, leading to cerebrospinal fluid leakage. In addition, cerebrospinal fluid leakage greatly increases the risk of intracranial infection and may lead to complications such as delayed wound healing. Recently, several experts have recommended the reposition of spinous process and lamina splitting after tumor resection, which could minimize spinal cord injury as much as possible and reduce the damage to spinal column stability, while sufficiently exposing the tumor. However, other problems, such as cerebrospinal fluid leakage, still cannot be properly resolved.

Hemi-laminectomy is another surgical approach that has gradually emerged. Compared with total laminectomy, this method has the advantages of minimally invasive surgery, preservation of surrounding muscles and the posterior column of the spine [21, 22]. Of note, this approach still has shortcomings such as cerebrospinal fluid leakage, intracranial infection, and damage to the attachment spots of the muscles on the operative side. In view of this, many spinal surgeons began to use minimally invasive expandable channel to perform tumor resection [23, 24]. However, the structure of traditional expandable channels is usually hollow cylinders. Therefore, the operative field depends on the diameter of the channels and for larger tumors, if operated on forcibly, it will greatly increase the risk of spinal cord and nerve damage [24]. In view of this, in order to expand the operative field under the premise of not expanding damage scope. Our group began to use a special instrument to form an inverted “V” shaped surgical access for tumor resection. In this study, the tumor removal rate was 100%. There were no cases of cerebrospinal fluid leakage or spinal instability. As for functional recovery, at the time of last follow-up, the median JOA score was significantly improved compared to the preoperative median JOA score. The overall excellent and good rate were 88.9%. The median VAS score at post-surgery was also significantly improved compared to the preoperative median VAS score. The results indicated that this technique is a safe and effective minimally invasive method. Contrasting with other methods reported before, our method is much safer and more effective with low complication rates [25, 26]. For example, Lee et al. [25] analyzed the clinical efficacy of 117 patients with intraspinal lesions receiving laminoplasty and the Leibinger mini-plate. In their study, the gross total resection was achieved in 82.9%, which was significantly lower than our result. Meanwhile, another study retrospectively analyzed the surgery outcomes of 101 patients with primary benign schwannoma. The average operation time in this study was 238 min, and the average estimated blood loss was 1275 mL. Compared with this study, our surgical method offers advantages of shorter operative time and lesser blood loss [26].

Based on our experience, schwannomas with a diameter less than 5 cm are suitable for this surgical approach. The advantages of this surgical approach are as follows: (1) By using our approach, the skin incision is small, whereas the internal surgical view is large. which could make the surgical field wider for operator. As for larger tumors, the operational range of inverted V-shaped access is much larger than traditional cannula, which could prevent insufficient exposure of the surgical field and inability to completely remove the larger tumor tissues. In our group, the maximum tumor diameter ranged from 2.1 to 5 cm, and should resolve the vast majority of cases. (2) Effectively avoid postoperative leakage of cerebrospinal fluid. In general, it is difficult to completely close the dural sac by simple suturing. In order to avoid cerebrospinal fluid leaking, we use an approach through the paraspinal muscles and sutures the deep fascia and superficial fascia, which effectively prevents the occurrence of cerebrospinal fluid leakage. (3) Compared with the pervious laminectomy surgery, this approach avoids damage to the spinous process and interspinous ligament, and effectively preserves the “three-column” structure of the spine, minimizes spinal damage, and maintains spinal stability.

Although this technique has many advantages over past methods, several surgical steps still require particular attention: (1) The surgeon’s knowledge of the relevant anatomy and microsurgical techniques should be proficient, and the intraoperative movements should be gentle to avoid causing spinal cord damage. (2) When the tumor is located in the thoracic or cervical spinal canal, the most important is not to forcefully remove the whole tumor tissue in one piece but to resect it in blocks. (3) When separating the tumor, the method of separation should be from the distal to the proximal end. For tumors located on the ventral or ventrolateral side of the spinal cord, the spinal cord should be pulled to the opposite side while using a tumor-hanging wire for traction [27]. (4) An accurate measurement of the tumor size before surgery is needed. And accurate localization of the tumor is also the key for successful surgery. (5) The inverted V-shaped surgical access can provide better surgical visualization and operating space for the surgeon. If it is still not enough, adjusting the angle of the operating table appropriately can achieve a better surgical view. (6) In terms of parent nerve root, in our opinion, there is no need to preserve parent nerve root at the cost of leaving residual tumor. In order to ensure a complete tumor resection and avoid tumor recurrence, parent nerve root is generally removed. Nevertheless, special care is taken to avoid damage adjacent nerve roots.

This study has several limitations, including being a retrospective analysis, having a small sample size, and lacking a comparative cohort. Therefore, further randomized controlled trials with larger samples is needed. In addition, the surgical instrumentation for the inverted V-shaped access is not specifically designed for orthopedic surgery, thus our group will develop the new surgical instrument for intraspinal schwannomas and also other spinal tumors.

## Conclusion

With the continuous development of minimally invasive spinal surgery technology, minimally invasive methods of spinal tumors have improved a lot. This study demonstrated a novel surgical technique, posterior paramedian approach combined with inverted V-shaped surgical access, as a minimally invasive method for the treatment of intraspinal schwannomas with excellent clinical results. To our understand, this approach can overcome the shortcomings such as large trauma and more complications in total laminectomy and could also provide sufficient surgical field exposure. Therefore, these advantages make this technique worthy of promotion and also provide inspiration for the future treatment of spinal tumors.

## Data Availability

The raw data of the current study are available from the corresponding author on reasonable request.
